# Supplementary material for: Reduction of Doxorubicin-Induced Cardiotoxicity by Co-Administration of Smart Liposomal Doxorubicin and Free Quercetin: In Vitro and In Vivo Studies
Source: Pharmaceutics. 2023 Jul 11;15(7):1920. doi: 10.3390/pharmaceutics15071920 (PMC10385381; doi:10.3390/pharmaceutics15071920)
Supplement: Supplementary file 1 [file pharmaceutics-15-01920-s001.zip › pharmaceutics-2394824-supplementary.pdf]

Supporting Information (SI)

Reduction of Doxorubicin-Induced Cardiotoxicity by Co-Administration of Smart Liposomal Doxorubicin and Free Quercetin: In Vitro and In Vivo Studies

Hamidreza Dorostkar [1], Bibi Fatemeh Haghiralsadat [2,3], Mahdie Hemati [1,3], Fatemeh Safari [4,5], Azam Hassanpour [6], Seyed Morteza Naghib [7,8], Mohammad Hossein Roozbahani [9], M. R. Mozafari [10] and Ali Moradi [1,*]

[1] Department of Clinical Biochemistry, Faculty of Medicine, Shahid Sadoughi University of Medical Sciences, Yazd 8916877391, Iran; hr.dorostkar@yahoo.com (H.D.); m.hemati1420@gmail.com (M.H.)
[2] Department of Advanced Medical Sciences and Technologies, Faculty of Paramedicine, Shahid Sadoughi University of Medical Sciences, Yazd 8916877391, Iran; fhaghirosadat@gmail.com
[3] Medical Nanotechnology and Tissue Engineering Research Center, Yazd Reproductive Sciences Institute, Shahid Sadoughi University of Medical Sciences, Yazd 8916877391, Iran
[4] Department of Physiology, Faculty of Medicine, Shahid Sadoughi University of Medical Sciences, Yazd 8916877391, Iran; fa.cardio@gmail.com
[5] Cardiovascular Research Center, Shahid Sadoughi University of Medical Sciences, Yazd 8916877391, Iran
[6] Department of Anatomical Sciences, Faculty of Medicine, Shahid Sadoughi University of Medical Sciences, Yazd 8916877391, Iran; azamhassanpour63@yahoo.com
[7] Nanotechnology Department, School of Advanced Technologies, Iran University of Science and Technology, Tehran 16846-13114, Iran; naghib@iust.ac.ir
[8] Biomaterials and Tissue Engineering Department, Breast Cancer Research Center, Motamed Cancer Institute, IUST, ACECR, Tehran 16846-13114, Iran
[9] School of Advanced Technologies, Iran University of Science & Technology, Tehran 16846-13114, Iran; roozbahani@iust.ac.ir
[10] Australasian Nanoscience and Nanotechnology Initiative (ANNI), Monash University LPO, Clayton, VIC 3168, Australia; dr.m.r.mozafari@gmail.com
* Correspondence: moradi20018@gmail.com; Tel.: +98-9126706056

The full (uncropped) Western blots given below are presented in full in the following pages of this Supplementary Document.

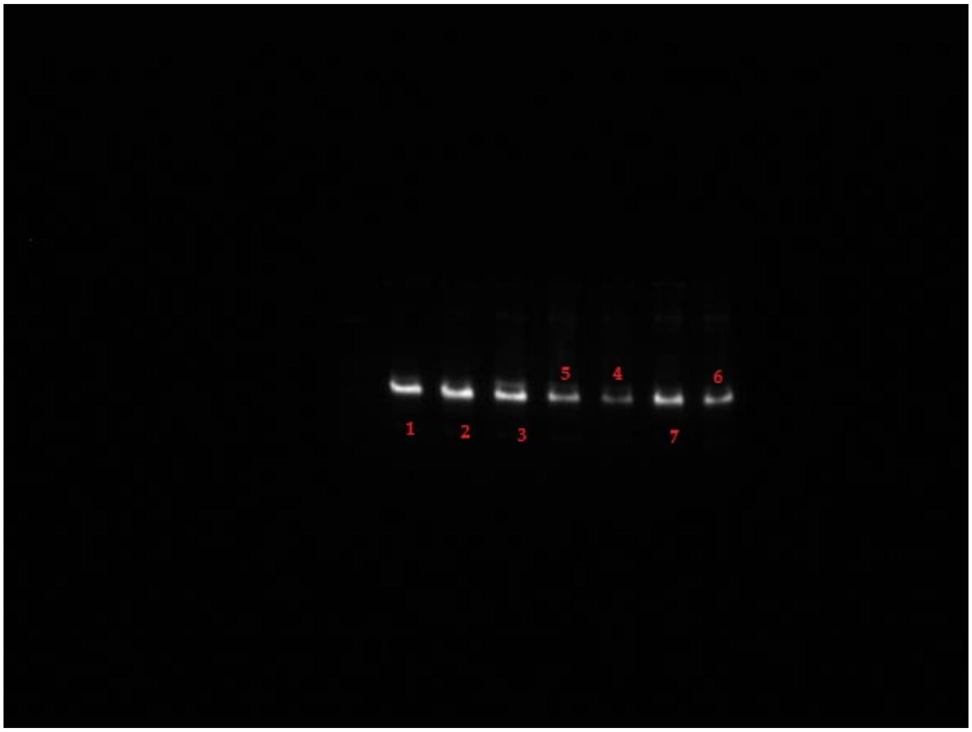

Figure SI.1.a. Western blot bands (Bcl2: ~ 26kDa)

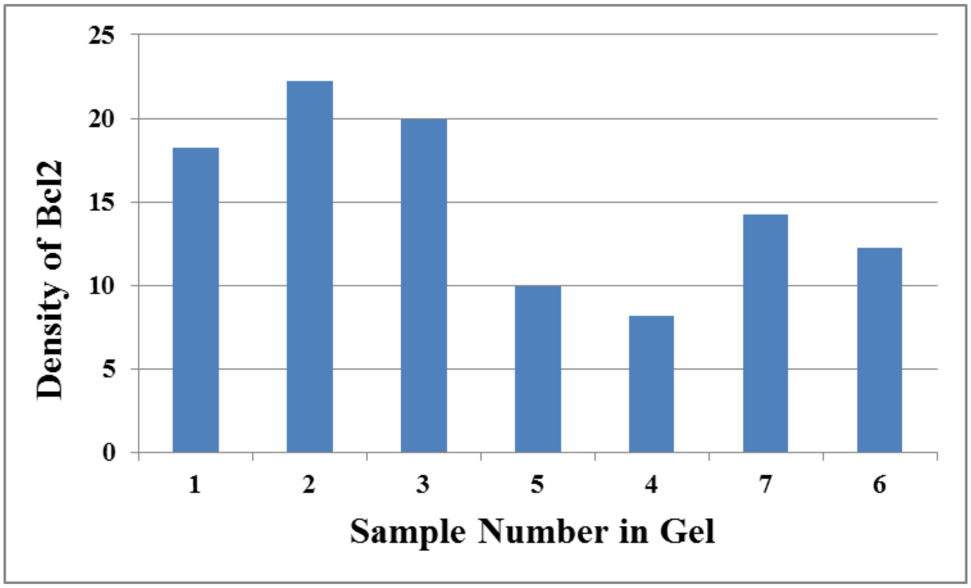

Figure SI.1.b. Densitometry of western blot bands (Bcl2)

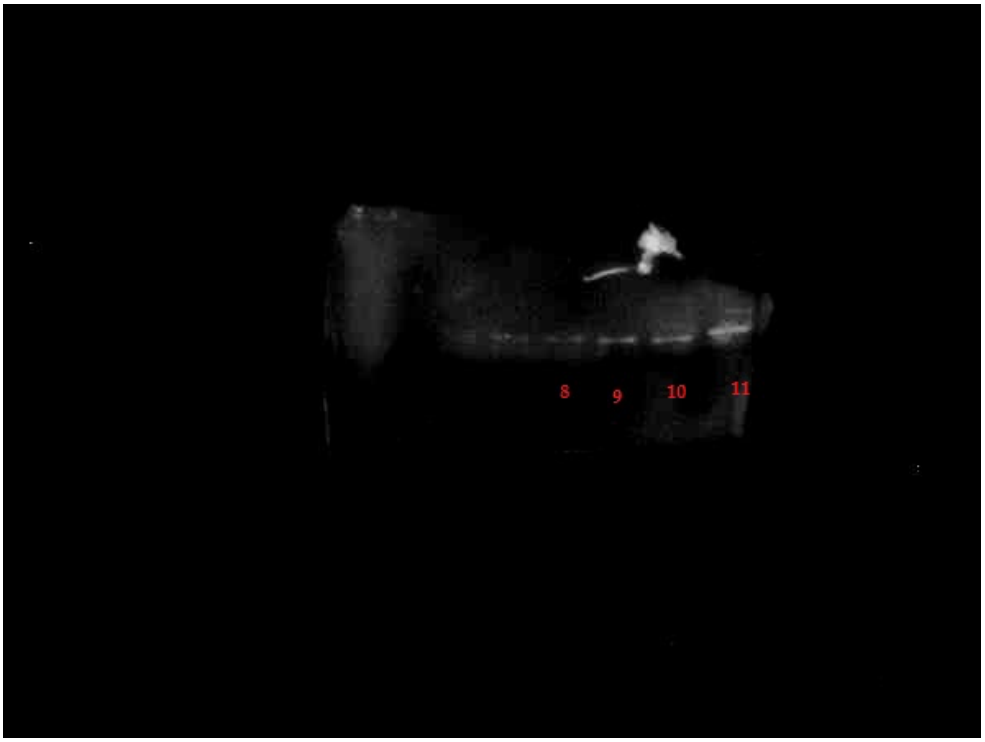

Figure SI.2.a. Western blot bands (NOX1: ~ 65kDa)

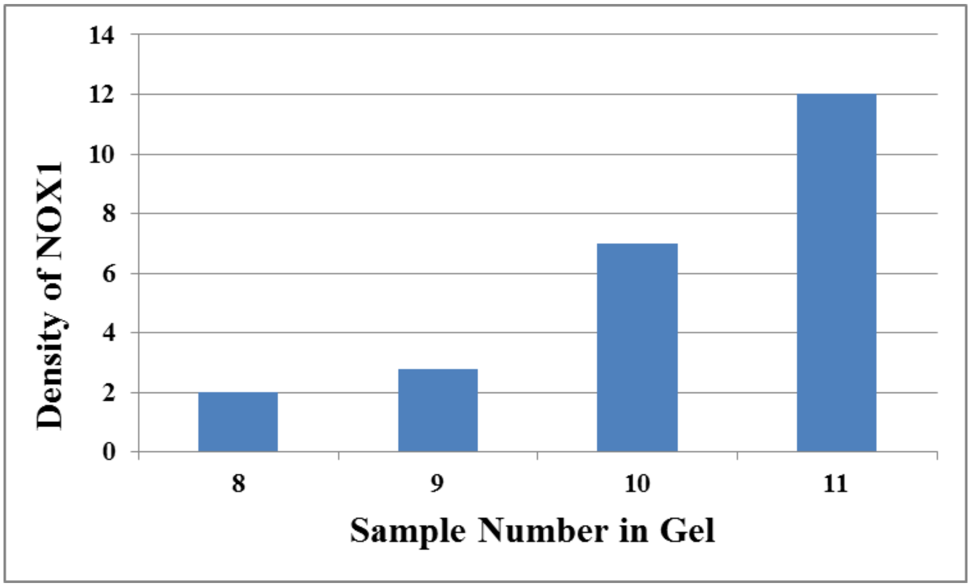

Figure SI.2.b. Densitometry of western blot bands (NOX1)

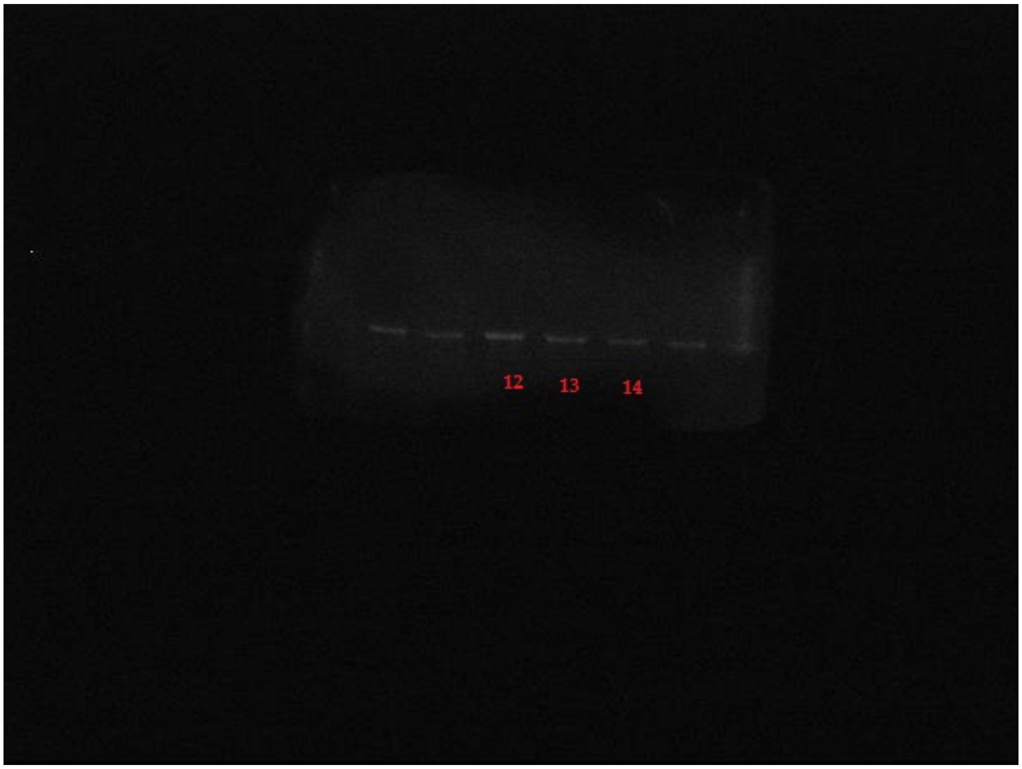

Figure SI.3.a. Western blot bands (NOX1: ~ 65kDa)

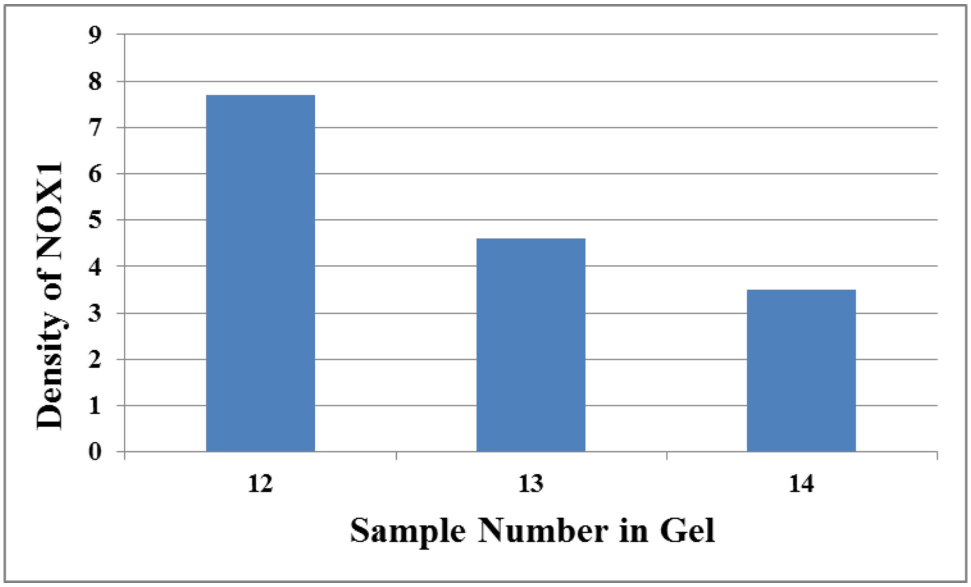

Figure SI.3.b. Densitometry of western blot bands (NOX1)

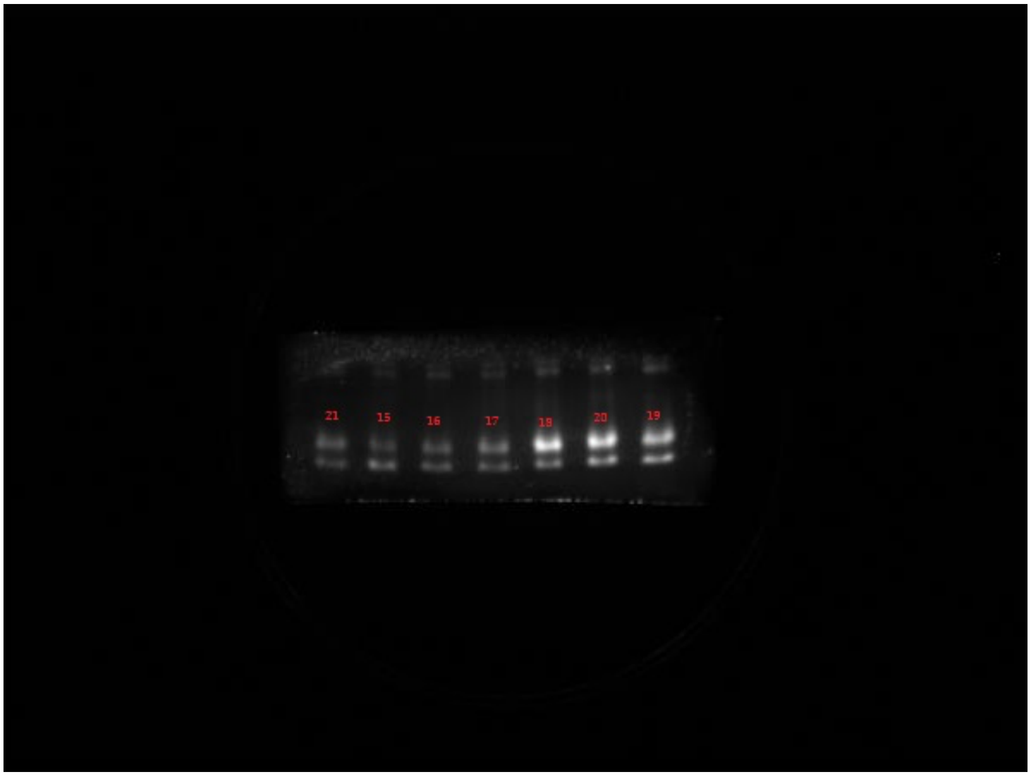

Figure SI.4.a. Western blot bands (Rac1: ~ 21kDa)

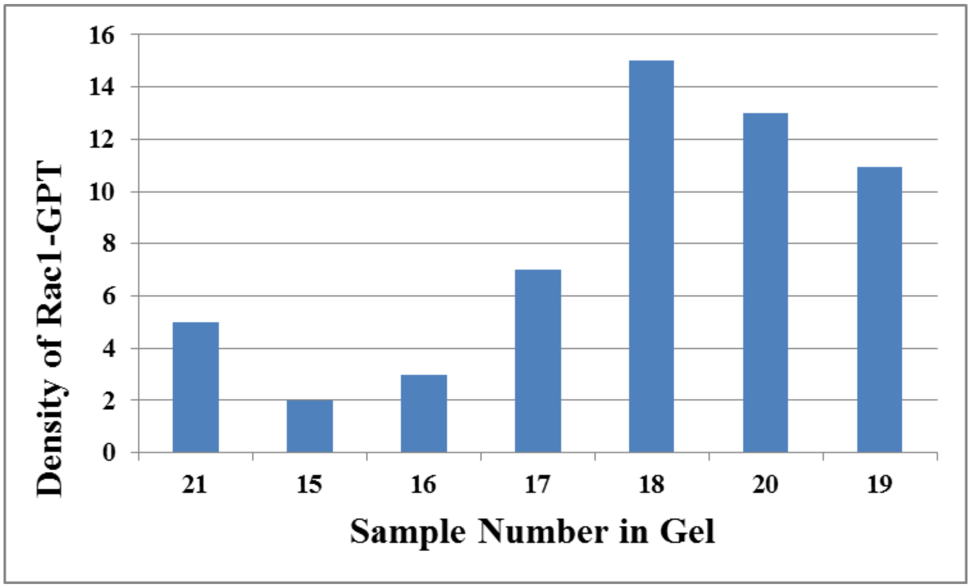

Figure SI.4.b. Densitometry of western blot bands (Rac1)

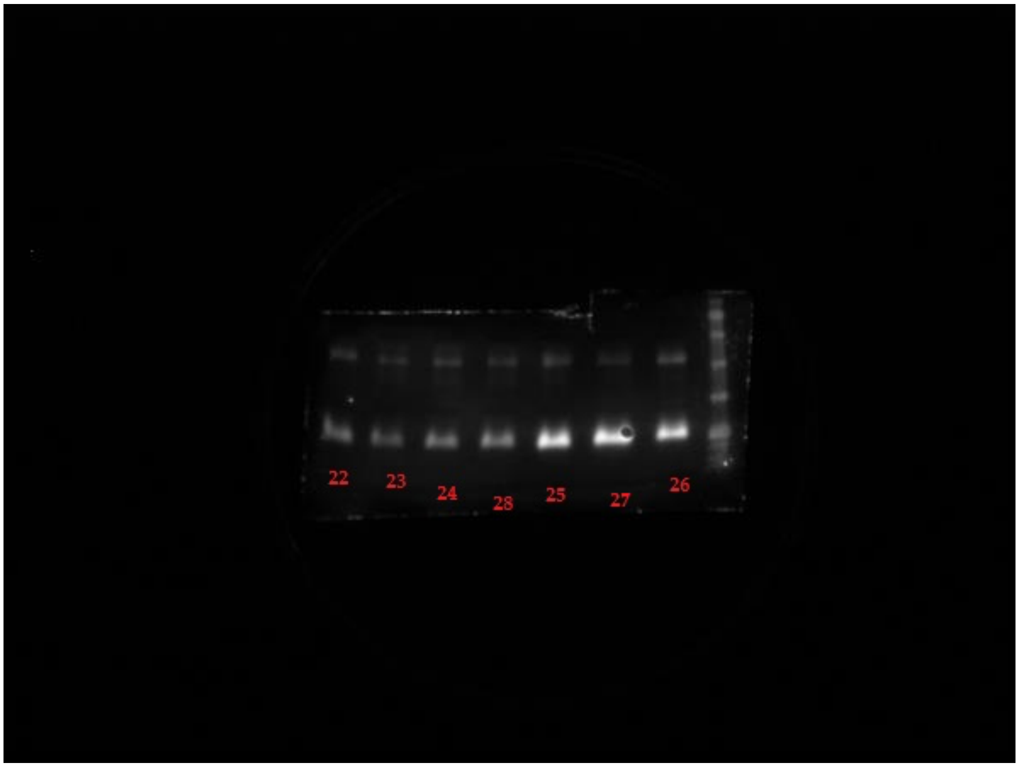

Figure SI.5.a. Western blot bands (Rac1-GTP: ~ 21kDa)

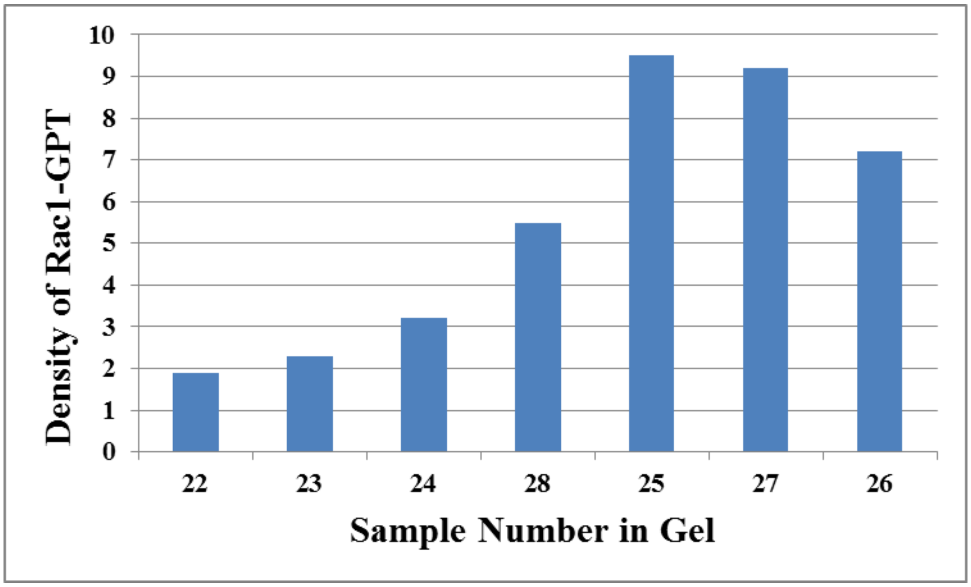

Figure SI.5.b. Densitometry of western blot bands (Rac1-GTP)

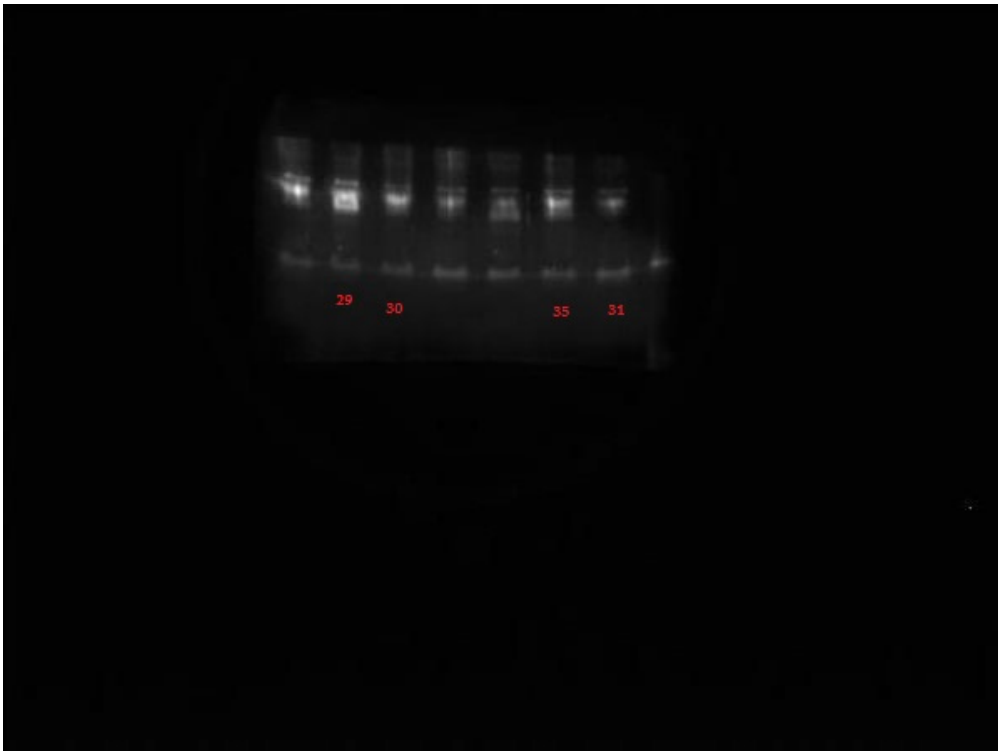

Figure SI.6.a. Western blot bands (SIRT3: ~ 28kDa)

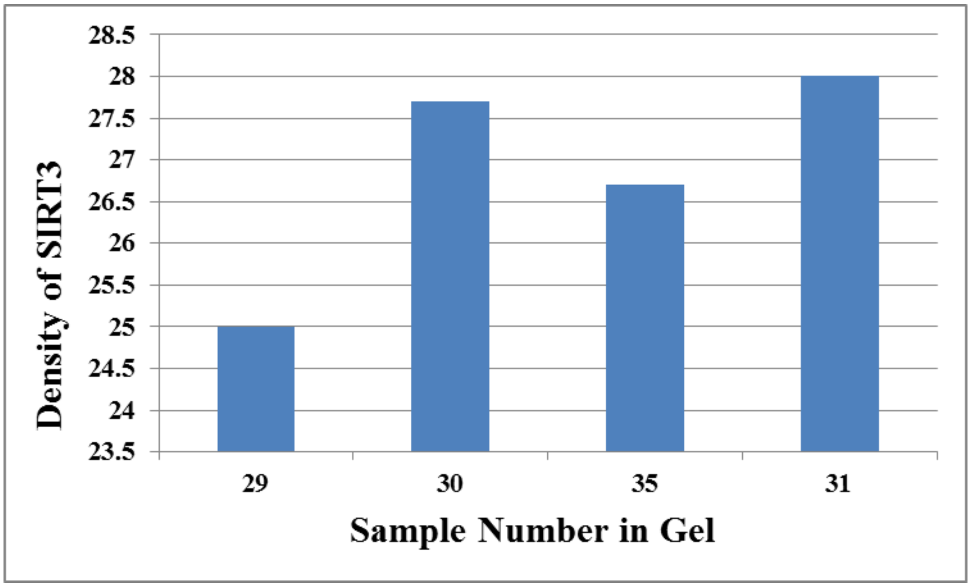

Figure SI.6.b. Densitometry of western blot bands (SIRT3)

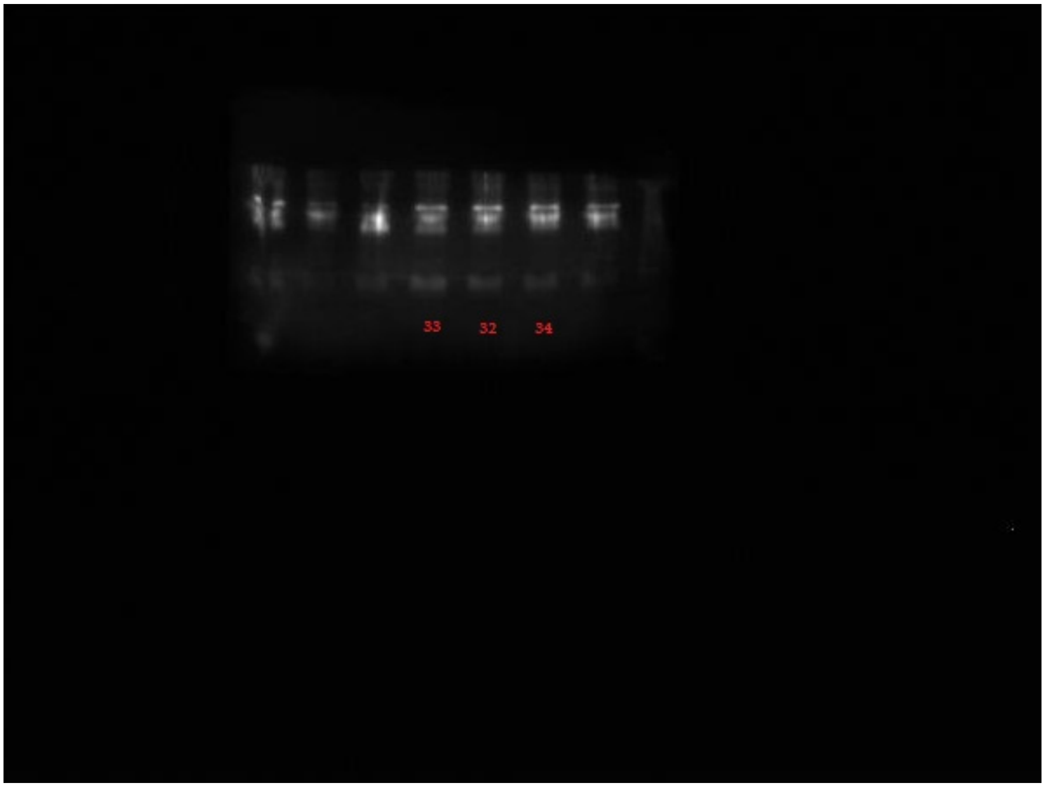

Figure SI.7.a. Western blot bands (SIRT3: ~ 28kDa)

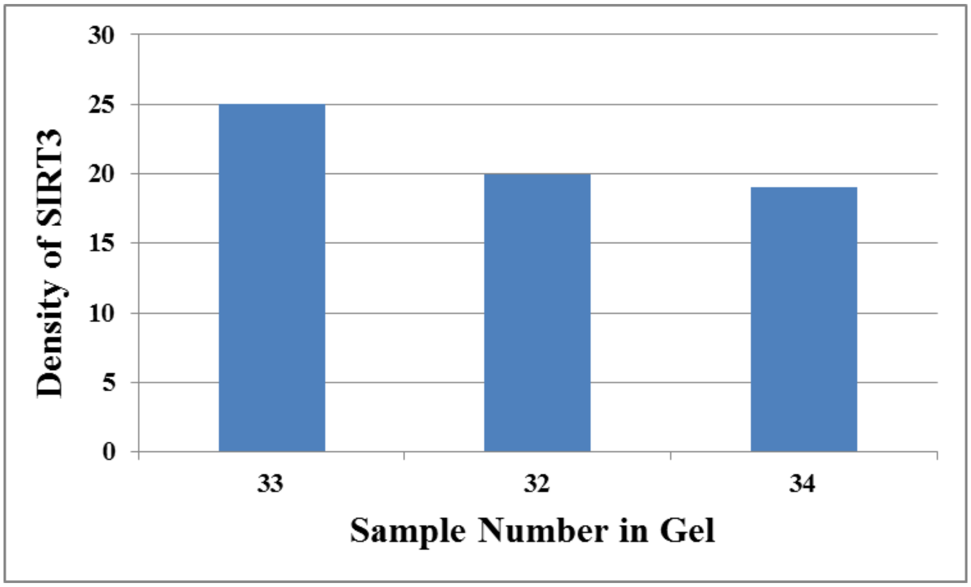

Figure SI.7.b. Densitometry of western blot bands (SIRT3)

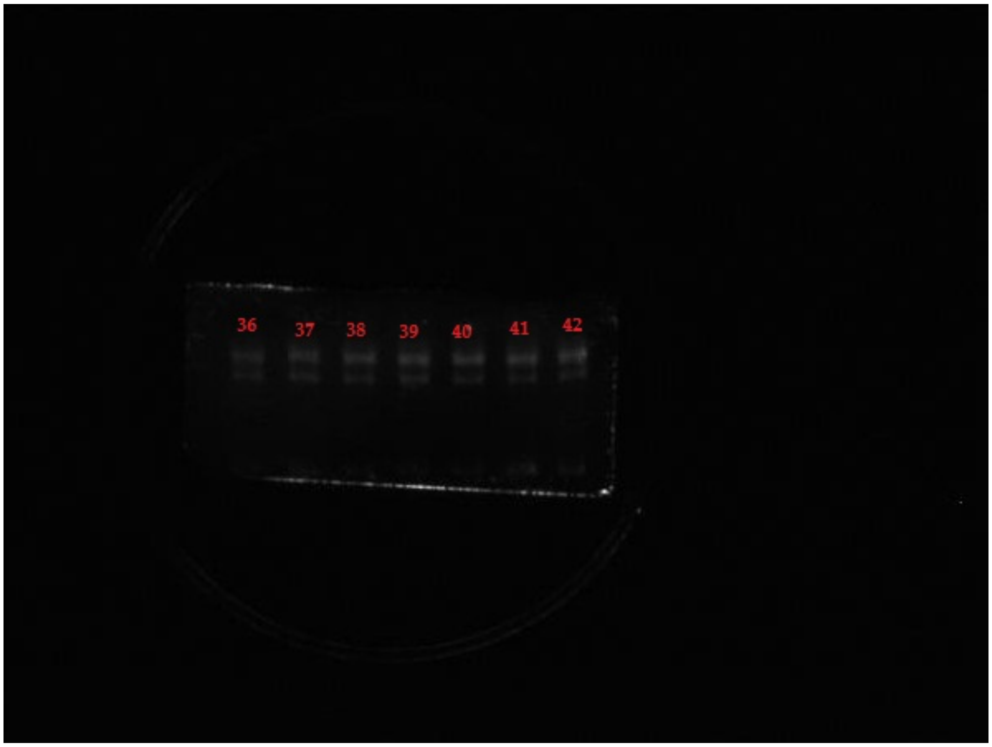

Figure SI.8.a. Western blot bands (b.Actin: ~ 42kDa)

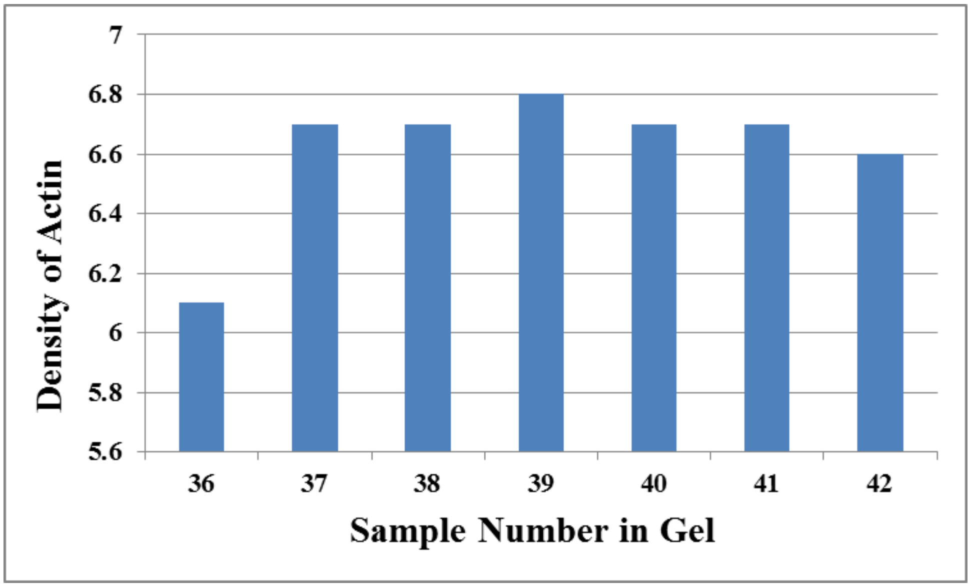

Figure SI.8.b. Densitometry of western blot bands (b.Actin)
